# A pretreatment ECM-high/immune-low transcriptomic score is associated with pathological complete response after neoadjuvant therapy in breast cancer

**DOI:** 10.1371/journal.pone.0357532

**Published:** 2026-11-05

**Authors:** Tao Wang, Xiaoyun Wu

**Affiliations:** 1 Department of Thyroid and Breast Surgery, Hubei No. 3 People’s Hospital of Jianghan University, Wuhan, Hubei, China; 2 Department of Cardiothoracic, Thyroid and Breast Surgery, Enshi Central Hospital, Enshi, Hubei, China; Longgang Otorhinolaryngology Hospital & Shenzhen Key Laboratory of Otorhinolaryngology, Shenzhen Institute of Otorhinolaryngology, CHINA

## Abstract

In this retrospective public-cohort observational study, we evaluated a biologically motivated extracellular matrix (ECM)-high/immune-low transcriptomic score for association with pathological complete response (pCR) after neoadjuvant therapy in breast cancer. The score contrasted standardized ECM/fibroblast and immune-response components. Cohort-specific logistic models adjusted for available clinical covariates; their performance was evaluated separately and was not validation of a locked transported model. In I-SPY2/GSE194040 (n = 987; 319 pCR events), a one-standard-deviation higher score was associated with lower pCR odds (odds ratio [OR] 0.687, 95% confidence interval [CI] 0.588–0.803). Inverse adjusted estimates were also observed in GSE25066 (OR 0.744, 95% CI 0.568–0.975) and GSE20271 (OR 0.407, 95% CI 0.232–0.715). Apparent areas under the receiver operating characteristic curve (AUCs) for these multivariable models were 0.750, 0.732 and 0.798; corresponding 500-bootstrap optimism-corrected estimates were 0.731, 0.713 and 0.765, conditional on processed scores. GSE20194 estimates were nonsignificant, and substantial record overlap with GSE25066 prevented interpretation as additional independent confirmation. A GSE25066 sensitivity restricted to deposited human epidermal growth factor receptor 2 (HER2)-negative status retained direction but had a confidence interval crossing unity. Higher scores correlated with lower immune-related reverse-phase protein array signals in matched I-SPY2 samples. The findings support a hypothesis-generating bulk-transcriptomic response association, with limited cross-cohort robustness. Heterogeneous treatment, sampling, assay and endpoint annotations, together with absent spatial and prospective validation, preclude claims of causality or clinical treatment-selection utility.

## Introduction

Neoadjuvant therapy in breast cancer can downstage disease and provide an early measure of treatment sensitivity. Pathological complete response (pCR) is associated with longer-term outcomes, with the strength of association varying by molecular subtype [1]. Interpretation of a pretreatment response marker therefore needs to account for the treatment and population in which it is evaluated.

Established genomic response studies already address several aspects of breast cancer treatment sensitivity. Hatzis et al. combined endocrine-sensitivity, chemotherapy-resistance and chemotherapy-sensitivity signatures and evaluated response and distant relapse-free survival in discovery and independent validation cohorts [2]. The I-SPY2 biomarker study combined immune, DNA-repair and human epidermal growth factor receptor 2 (HER2)/luminal phenotypes to investigate response-based treatment subtypes across ten regimens [3]. Dugo et al. assessed the 27-gene DetermaIO assay in the randomized NeoTRIP comparison of chemotherapy with or without atezolizumab in triple-negative breast cancer [4]. Our analysis reuses public data from the I-SPY2 and other neoadjuvant research settings; it does not introduce a new cohort or demonstrate superior performance to those predictors.

The biological rationale also has substantial precedent. Ayers et al. linked interferon-related and T-cell-inflamed expression programs to pembrolizumab response across solid tumors [5]. Mariathasan et al. associated fibroblast transforming growth factor β (TGF-β) signaling with poor programmed death-ligand 1 (PD-L1)-blockade response in metastatic urothelial cancer and tested combined blockade in mouse models [6]. Breast cancer studies have investigated collagen-associated macrophage regulation of CD8 + T-cell function [7] and extracellular matrix (ECM)-producing fibroblasts in stromal fibrosis and immune exclusion [8]. These studies motivate examination of matrix and immune programs, but they do not establish the present score as a spatial measurement or as a predictor of benefit from a particular neoadjuvant regimen.

Liu et al. derived an 11-gene ECM index from The Cancer Genome Atlas (TCGA) survival data using Cox regression and least absolute shrinkage and selection operator (LASSO) methods. Separately, they selected pCR/residual disease (RD)-associated genes and fitted a neural network using merged GSE16446, GSE32642, GSE50948 and GSE66399 data, with random training/testing division [9]. Here, two fixed equal-weight components are contrasted without fitting their gene weights to pCR; response associations are estimated separately in I-SPY2, GSE25066 and GSE20271, with overlapping GSE20194 data treated as auxiliary evidence. This is a focused association assessment, not a first ECM–immune concept or a comparative superiority study.

Cancer-associated fibroblasts (CAFs) are heterogeneous and may have context-dependent functions [10]. A bulk ECM-high/immune-low contrast therefore offers a testable summary of expression, while its interpretation remains sensitive to tissue composition, sampling, treatment and assay processing. Its simplicity makes the score calculation explicit, but does not by itself establish temporal prespecification or clinical transportability.

We evaluated biologically motivated components without estimating gene weights from pCR and evaluated the score separately in the main cohort and each external dataset. This approach does not seek to rediscover an optimized multigene classifier. Instead, it tests whether an interpretable ECM-high/immune-low signal is directionally and quantitatively associated with pCR after adjustment for variables available within each dataset. The GSE20194 analyses are reported as auxiliary or sensitivity evidence. Revision-stage auditing identified substantial record overlap with GSE25066, including 92 of the 100 MAQC validation samples; these analyses therefore cannot be counted as additional independent validation. We hypothesized that tumors with higher scores would have lower pCR odds after neoadjuvant therapy. I-SPY2/GSE194040 was the main cohort, GSE25066 and GSE20271 were primary external cohorts, GSE20194 was used for auxiliary and sensitivity analyses, and I-SPY2 reverse-phase protein array (RPPA) data provided protein-level context [3,11]. We report this study in accordance with the Strengthening the Reporting of Observational Studies in Epidemiology (STROBE) reporting checklist.

## Materials and methods

### Study design and public cohorts

This retrospective analysis included public treatment-naive breast cancer transcriptomic cohorts with neoadjuvant response endpoints. I-SPY2/GSE194040 was the main cohort [3]. GSE25066 and GSE20271 were analyzed as external response cohorts. The dataset hierarchy was developed during exploratory feasibility work and should not be interpreted as a prospectively registered validation plan. GSE20194 is reported as overlapping auxiliary evidence, including its original MAQC subsets and exclusion sensitivities. I-SPY2 RPPA data from GSE196093 provided protein-level context [11]. I-SPY2 expression samples were matched to clinical annotations using platform-aware sample identifiers. One patient had two platform-specific pretreatment samples; this duplicate was resolved using a GPL20078-priority rule documented in the analysis code.

### Sample matching and duplicate-platform sensitivity

The expression matrix contained 988 sample columns. Platform-aware identifiers matched all 988 columns to public clinical records, representing 987 unique patients. One patient had two pretreatment platform records; the original code retained the GPL20078 record. This is documented as an implemented processing rule, without claiming that the platform choice was prospectively registered or proven superior. The expression-to-patient manifest preserves both records, source platform labels, outcome annotations, inclusion status and exclusion reason. As a revision-stage sensitivity analysis, we replaced the retained score with the alternate-platform score, retained the clinical covariates, restandardized the final patient-level score and refitted the same model. The adjusted odds ratio (OR) was 0.68705 (95% confidence interval [CI] 0.58807–0.80268), compared with 0.68748 (95% CI 0.58844–0.80319) under the original rule.

### ECM–immune exclusion score

Expression values were processed within each cohort. I-SPY2 used the provided gene-level expression matrix. GPL96 external cohorts were mapped from probe to gene symbols using the GPL96 annotation file; when multiple probes mapped to the same gene, the probe with the largest interquartile range was retained. For this implemented probe-selection rule, IQR was computed on log2(x + 1) values in GSE20271, and in GSE20194/GSE25066 only when the maximum source expression value exceeded 100; otherwise the source scale was used. After selecting a probe, the original deposited expression values, rather than this temporary IQR-scale matrix, were used for score calculation. Gene-level values were converted to row-wise z scores: each gene was centered by its mean and divided by its sample standard deviation across expression records within the same cohort, before component averaging. Means and standard deviations used nonmissing values. A gene with zero or undefined standard deviation was assigned missing z scores and did not contribute to the available-gene component mean; none of the score genes available on the four cohort platforms had zero or undefined standard deviation. The ECM/CAF/TGF-β score was calculated as the mean z-score expression of *COL1A1*, *COL1A2*, *COL3A1*, *COL5A1*, *COL5A2*, *COL6A1*, *COL6A2*, *COL6A3*, *FN1*, *POSTN*, *THBS2*, *SPARC*, *MMP2*, *MMP11*, *LOX*, *LOXL2*, *ACTA2*, *FAP*, *PDGFRB*, *TAGLN*, *VIM*, *TGFBI*, *TGFB1*, *SERPINE1*, *CTGF*. The interferon γ (IFN-γ)/cytotoxic/antigen-presentation score was calculated as the mean z-score expression of *IFNG*, *CXCL9*, *CXCL10*, *STAT1*, *IRF1*, *IDO1*, *GZMA*, *GZMB*, *PRF1*, *NKG7*, *GNLY*, *CD8A*, *CD8B*, *CD3D*, *CD3E*, *HLA-A*, *HLA-B*, *HLA-C*, *B2M*, *TAP1*, *TAP2*, *PSMB8*, *PSMB9*, *NLRC5*, *LAG3*, *PDCD1*, *CD274* when available. At the platform level, ECM gene-set coverage was 25/25 in all cohorts; immune-component coverage was 27/27 in I-SPY2 and 25/27 in the GPL96 cohorts because *NLRC5* and *CD274* were unavailable. These components operationalize matrix/fibroblast and immune-response programs. Prior literature supports this broad biological rationale [5,10], but does not validate this exact gene combination, weighting or subtraction rule. The inspected score-construction scripts use fixed lists and equal weights without outcome-based fitting; the authors confirm that the gene lists were manually curated and fixed before examining the results of the present study and were not subsequently modified in response to those results. The final ECM-immune exclusion score was defined as z(ECM/CAF/TGF-β score) minus z(IFN-γ/cytotoxic/antigen-presentation score). Gene and component standardization used 988 I-SPY2 expression records, 508 GSE25066 records, 178 GSE20271 records and 278 GSE20194 records, before outcome or covariate exclusions. The final contrast was restandardized for regression on 987 unique, endpoint-usable I-SPY2 patients after the upstream outcome/score eligibility check and duplicate-platform resolution; in GSE25066, it was standardized on all 508 processed records before the outcome and covariate exclusions that yielded 488 endpoint-usable and 482 model-complete patients. The corresponding final-score standardization populations were 178 in GSE20271 and 278, 230 and 100 for the GSE20194 all-record, training-plus-validation and validation-only analyses, respectively. For GSE20194, subset selection preceded final-score standardization; gene and component normalization still used the full 278-record expression cohort. Final scores were not restandardized after model-specific exclusions. ORs therefore represent the association per one-standard-deviation (SD) higher score in the stated standardization population, rather than necessarily one SD among the complete cases. In this manuscript, ECM-immune exclusion denotes a bulk transcriptomic ECM-high/immune-low state rather than direct spatial immune exclusion.

### Specification history and analysis status

The score combines fixed equal-weight expression averages for 25 ECM-related and 27 immune-related genes. The authors confirm that these gene lists were manually curated and fixed before examining the results of the present study and were not subsequently modified in response to those results. The inspected construction scripts do not estimate gene weights or select genes by pCR. However, we have not identified a dated preregistration or immutable repository record establishing that every preprocessing choice, model and cohort hierarchy was fixed before feasibility analyses. We therefore describe the score as biologically motivated and the study as retrospective and exploratory. The cohort hierarchy was refined during feasibility work; the nonsignificant GSE20194 analyses and all revision-stage sensitivity results are retained. The additional analyses requested during peer review are labeled as revision-stage sensitivity or internal-validation analyses rather than prospectively confirmatory tests.

Among the 987 selected I-SPY2 samples, 967 had nonmissing expression for all 25 ECM and 27 immune genes; 16 had 25/26, three had 24/27 and one had 24/26 nonmissing genes. Component means used the available genes in each sample, so sample-level denominators could be lower than platform coverage. Missing expression was not replaced with zero.

### Outcome and covariates

The primary endpoint was pCR, coded as a binary variable from cohort-specific response annotations. I-SPY2 used the pcr annotation from Gene Expression Omnibus (GEO) series metadata, GSE25066 used pathologic_response_pcr_rd, GSE20271 used pcr_or_rd, and GSE20194 used pcr_vs_rd. The biologically motivated gene sets, detailed outcome coding, and missingness are provided in S1 Table and S2 Table. Models used complete cases for the score, pCR, and included covariates. In I-SPY2/GSE194040, the main adjusted model included hormone receptor status, HER2 status, MammaPrint risk, and treatment arm; a naming variant in one treatment arm label was harmonized before modeling. In GSE25066, the adjusted model included estrogen receptor status, clinical stage group, and study source. In GSE20271, the adjusted model included estrogen receptor status, HER2 status, and treatment. In GSE20194, the auxiliary adjusted model included estrogen receptor and HER2 status. Sparse treatment-code adjustment was not used for the main auxiliary estimate because several treatment levels had very small counts.

### Source endpoint definitions and comparability

I-SPY2 defines pCR as ypT0/is ypN0, but its modified intention-to-treat non-pCR category can include progression, treatment switching, withdrawal or no surgery [3]. The GSE20271 publication defines absence of invasive breast and nodal cancer and also classifies clinical progression as RD [12]. The GSE20194 Series description gives an invasive breast-and-node endpoint for its documented 230-patient MAQC subset; it does not establish complete pathology comparability for every additional record. These definitions allow residual in-situ disease in principle, but individual residual ductal carcinoma in situ and isolated-cell annotations are unavailable. The deposited I-SPY2 clinical annotations used here provide a binary pCR indicator without separate variables for progression, treatment switching, withdrawal or absence of surgery. Consequently, the numbers in these non-pCR categories are unavailable and cannot be reported separately. We preserve the source labels and cannot reconstruct a uniformly pathology-confirmed endpoint for all patients.

The original response fields map pCR (or I-SPY2 value 1) to 1 and RD (or I-SPY2 value 0) to 0. Twenty GSE25066 records without a usable response remain excluded. We did not replace pCR/RD with its combined residual cancer burden (RCB)-0/I category: among 118 RCB-0/I records, 80 have a pCR label and 38 retain RD; another 19 pCR records have no RCB category. The full mapping and cross-tabulation are included in S2 Table. A source-definition restriction considers I-SPY2, GSE20271 and the documented GSE20194 MAQC subset separately; it is an evidence restriction, not a new pooled model or independent replication.

Adjustment sets reflect available covariates rather than a common treatment-independent estimand. Source treatment schedules, dates and sampling methods differ. GSE25066 includes patients whose full chemotherapy course was not entirely preoperative [2]; individual timing cannot be recovered reliably for every analyzed record. No source-wide treatment assignment was inferred from missing individual metadata.

### Ethics statement and data access

This study was a secondary analysis of publicly available, de-identified research data deposited in the NCBI Gene Expression Omnibus (GEO): GSE194040 and GSE196093 (within the I-SPY2 SuperSeries GSE196096), GSE25066, GSE20271 and GSE20194. We used the deposited expression, protein and clinical annotations; public study/sample identifiers were used to match research records. No directly identifying participant information was used. The study involved no new participant recruitment or contact, clinical intervention, or collection of human specimens.

No additional ethical review was required for this secondary analysis under Article 32(1) of China’s Measures for the Ethical Review of Life Science and Medical Research Involving Humans (2023), which permits exemption for qualifying research using legally obtained public data. The present analysis was limited to de-identified public research records, involved no physical intervention and was undertaken for academic research. This exemption rationale concerns the present secondary analysis and does not constitute a claim that a new institutional ethics approval or written waiver was issued. Ethics and consent arrangements for the original collection of data belong to the originating studies; source-specific information and documentation limits are reported in S2 Table.

### Statistical analysis

Logistic regression estimated the relationship between the ECM-immune exclusion score and pCR. The score was analyzed as a continuous standardized variable, and odds ratios (ORs) were reported with 95% confidence intervals (CIs). Covariate availability differed across cohorts, so adjusted models were specified separately for each dataset. The area under the receiver operating characteristic (ROC) curve (AUC), apparent calibration, and decision-curve summaries describe within-cohort performance rather than external validation of a single transportable prediction model. Model discrimination was summarized by AUC and average precision (AP). Original apparent AUC 95% confidence intervals used the DeLong method through pROC::ci.auc on unsmoothed full ROC curves (pROC 1.18.5); these intervals are distinct from the revision-stage bootstrap optimism correction. Brier score, apparent calibration intercepts, apparent calibration slopes, and decision-curve net benefit were calculated as descriptive summaries from the fitted models, not as prospectively validated clinical utility estimates [13]. Interaction tests used likelihood-ratio tests comparing models with and without score-by-factor interaction terms. These analyses remain exploratory. In the revision, we applied Benjamini-Hochberg adjustment across all 15 reported cohort-factor tests; the nominal MammaPrint and GSE20271 treatment interactions each had q = 0.263 and did not provide multiplicity-adjusted evidence of effect modification. RPPA associations were assessed with Spearman correlation. False-discovery rates were adjusted using the Benjamini-Hochberg method within each score-specific family of protein-marker tests. Analyses were executed using reproducible R scripts with set.seed(123); package versions and thresholds are archived with the submission materials.

### Revision-stage validation and sensitivity methods

Study size was determined by the available public expression and endpoint records, not by a prospective sample-size calculation. We report event counts, model degrees of freedom and confidence intervals rather than retrospective observed power. The six selected models used 16, 6, 4, 3, 3 and 1 slope parameters, respectively. GSE20271 therefore had 6.5 pCR events per slope parameter. Such ratios are descriptive warnings about precision, not proof of model adequacy.

We inspected convergence, design-matrix rank, coefficient/standard-error finiteness and fitted-probability ranges. A separate linear-programming screen evaluated whether any coefficient direction classified all outcomes with nonnegative signed margins and at least one positive margin. The bounded-coefficient optimization used HiGHS through SciPy 1.17.1, feasibility tolerance 10^−9^ and detection tolerance 10^−7^; known complete, quasi-complete and overlapping examples verified sign conventions. This numerical diagnostic applies to the original selected designs, not every possible bootstrap sample or model specification.

In an additional revision-stage analysis, we used 500 nonparametric patient-level bootstrap samples within each reported cohort analysis set. Each bootstrap sample refitted the same regression formula; its performance was evaluated in both the bootstrap sample and the original analysis set. Optimism was the average difference between these evaluations, and the corrected estimate was the original apparent performance minus optimism. ROC direction was fixed so that a larger predicted probability indicated pCR. This procedure was conditional on the observed expression-derived scores and cohort preprocessing; it did not repeat raw-expression preprocessing, assess a locked model in a new cohort, or establish clinical utility.

We fitted ridge-penalized logistic models retaining the original score and covariates, with lambda fixed at 1/n and 10/n for this revision-stage sensitivity analysis. In glmnet, alpha was 0, lambda was the total penalty divided by the analysis sample size, the intercept was unpenalized, and predictors were not restandardized. We did not select genes or optimize a penalty to obtain favorable associations. Conditional percentile intervals were obtained from 500 patient bootstrap samples per model and penalty. These shrinkage-based intervals are supplementary and do not replace the original unpenalized association estimates.

ER imputation in GSE25066 used 50 chained-equation logistic imputations, 10 iterations and pCR, score, stage and source as predictors. Only the six missing ER values among endpoint-usable patients were imputed; outcome labels were not imputed. Coefficients and uncertainty were pooled with mice::pool under Rubin’s rules. The conditional missing-at-random assumption remains untestable from observed data.

For apparent calibration, a logistic natural-spline model with three degrees of freedom related observed pCR to the original model logit. Curves were shown over the 2.5th–97.5th percentiles of fitted probabilities, with 500 patient-bootstrap pointwise percentile bands conditional on the original predictions. Bands are not simultaneous and do not include full model-development uncertainty. Decision curves used thresholds 0.05–0.50 at 0.05 increments, with mathematical treat-all and treat-none comparators. Net benefit equaled true positives/n minus false positives/n multiplied by threshold/(1 − threshold). The same paired bootstrap optimism subtraction was applied to net benefit. Thresholds are illustrative; no validated treatment decision, action cost or clinical utility is claimed.

All 139 score–RPPA correlations are reported with marker-specific n, nominal P, BH q and 500-resample percentile intervals. BH adjustment also covers the family of all 15 reported interaction tests. Primary associations, auxiliary analyses and every revision-stage sensitivity are listed in the analysis inventory; none is upgraded to prospectively confirmatory evidence. The other sensitivity procedures address source-label/profile overlap, probe aggregation, rank normalization, missing genes, duplicate platforms, receptor definitions, treatment assignment/crossovers and source endpoint/eligibility restrictions. Scripts retain all reported estimates, including attenuated or nonsignificant results.

Apparent calibration curves with conditional bootstrap bands are shown in S1 Fig, and illustrative optimism-corrected decision curves are shown in S2 Fig. The complete model coefficients, apparent metrics, diagnostics and internal-validation outputs are supplied in S3 Table; clinical-model comparisons are in S4 Table. Exploratory interactions and subgroups are in S5 Table, and decision-curve estimates with numerical warnings are in S6 Table. Figure-level data and all protein-marker results are in S7 Dataset. S8 Code supplies the analysis code, input hashes and reproduction instructions. Independent reconstruction from the raw files reproduced 53 computed TSV outputs within absolute/relative tolerance 10^−10^; three supplied curation/reporting documents were tracked separately. All nine remotely retrieved source files matched the archived input hashes. The analysis inventory and sensitivity results, including attenuated findings, are in S9 Table.

### Use of artificial intelligence tools

The authors used Codex for language polishing of the manuscript. No AI tools were employed in other parts of the study, including study design, data collection, analysis, figure preparation, and conclusion formulation. All authors have reviewed and verified the AI-generated outputs, and take full responsibility for the content of this manuscript.

## Results

### Cohort composition

The main analysis included 987 endpoint-usable I-SPY2/GSE194040 patients, including 319 pCR events. The selected adjusted model also included 987 complete cases. The primary external cohorts included 488 endpoint-usable GSE25066 patients with 99 pCR events and 178 GSE20271 patients with 26 pCR events. Complete-case adjusted models included 482 patients with 98 pCR events in GSE25066 and 178 patients with 26 pCR events in GSE20271. GSE20194 contributed auxiliary analyses in the all-sample set and in the published training-plus-validation and validation-only subsets (Table 1).

**Table 1:** Analysis cohorts and sample flow.

Dataset	Role	Entry n	Endpoint n (pCR)	Selected model n (pCR)	pCR rate	Notes
I-SPY2	Main	987	987 (319)	987 (319)	319/987 (32.3%)	Treatment-naive mRNA; adjusted for HR, HER2, MammaPrint, and treatment arm.
GSE25066	Primary external	508	488 (99)	482 (98)	99/488 (20.3%)	Largest external GPL96 cohort; adjusted for ER, stage, and study source.
GSE20271	Primary external	178	178 (26)	178 (26)	26/178 (14.6%)	Fine-needle aspiration cohort; adjusted for ER, HER2, and treatment.
GSE20194 all	Auxiliary	278	278 (56)	278 (56)	56/278 (20.1%)	Full cohort retaining published MAQC structure.
GSE20194 train+valid	Auxiliary	230	230 (48)	230 (48)	48/230 (20.9%)	Published MAQC training-plus-validation subset.
GSE20194 validation	Sensitivity	100	100 (15)	100 (15)	15/100 (15.0%)	Imprecise univariable estimate; not independent confirmation.

Notes: pCR, pathological complete response; HR, hormone receptor; ER, estrogen receptor; HER2, human epidermal growth factor receptor 2; MAQC, MicroArray Quality Control. I-SPY2 entry n is the patient count after matching 988 expression records and resolving one duplicate. External entry counts refer to scored profiles; GSE20194 subset entries reflect their source-defined membership. GSE20194 rows are nested analysis sets and must not be summed.

Fig 1 displays the cohort flow and pCR-event counts across these nested analysis sets.

**Fig 1:**
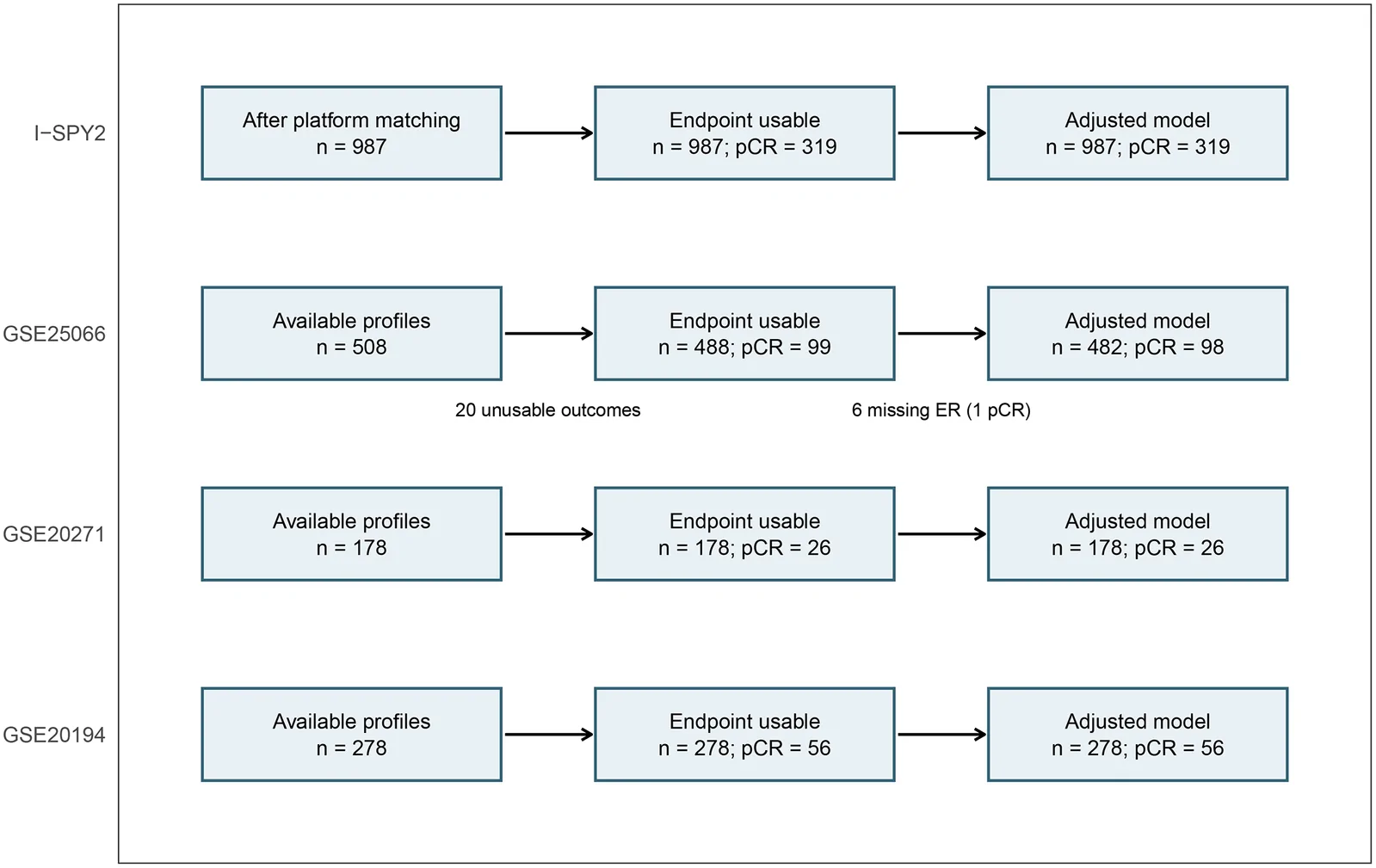
Cohort inclusion and selected-model samples. Boxes show processed, endpoint-usable and complete-case counts. I-SPY2 entry follows platform matching and duplicate resolution. GSE20194 subsets are nested and substantially overlap GSE25066; they must not be counted as independent additional cohorts.

### Component distributions across platforms

S3 Fig displays both component distributions and their joint relationships for all scored expression records, before outcome and covariate exclusions. In I-SPY2, the 654 GPL20078 and 334 GPL30493 records had ECM–immune Spearman correlations of −0.214 and −0.262, respectively; both records of the duplicated patient are included in this assay-level description. Corresponding correlations were −0.005 in GSE25066 (508 records), 0.036 in GSE20271 (178) and −0.045 in GSE20194 (278). These are mean gene-wise z-score components before component standardization. I-SPY2 platform groups share the original normalization, whereas external cohorts use cohort-specific normalization. The distributions describe the inspected data and cannot establish assay interchangeability or a universal reference for an individual patient (S7 Dataset; S9 Table).

### Descriptive clinical characteristics

Among endpoint-usable samples, I-SPY2 had 535/987 HR-positive and 245/987 HER2-positive cases. ER-positive counts were 285/488 in GSE25066 (six unknown), 98/178 in GSE20271 and 164/278 in GSE20194. Source HR status is not interchangeable with ER alone. Median age was 49 years in the two cohorts with inspected age fields; age and stage unavailable in other processed extracts were not inferred from source averages. The complete source, clinical-category and missingness tables are supplied in S2 Table, with endpoint-usable and adjusted-model denominators separated.

For descriptive interpretation, cohort-relative terciles were formed using the one-third and two-thirds quantiles of the final standardized score among patients with a nonmissing binary pCR label and a finite score, without requiring complete covariates. The quantiles used R type 7 linear interpolation. The cutpoint populations were I-SPY2 (n = 987), GSE25066 (n = 488), GSE20271 (n = 178), GSE20194 all records (n = 278), training plus validation (n = 230) and validation only (n = 100). In I-SPY2, the cutpoints were −0.449680 and 0.527968: low scores were at or below the first cutpoint, middle scores were above the first and at or below the second, and high scores were above the second. This right-closed grouping rule was used in every analysis set; exact cutpoints and their calculation populations are provided in S2 Table (score_tercile_cutpoints.tsv). I-SPY2 pCR frequencies were 144/329 (43.8%) in the low-score group and 79/329 (24.0%) in the high-score group. S2 Table reports all tercile counts and exact binomial intervals. These are unadjusted summaries, not a validated clinical cutoff or an adjusted treatment effect.

### Main association with pCR

In I-SPY2/GSE194040, each 1-SD higher ECM-immune exclusion score was associated with reduced pCR odds after adjustment for hormone receptor status, HER2 status, MammaPrint risk, and treatment arm (OR=0.687, 95% CI = 0.588–0.803, *P* < 0.001). The selected multivariable model had an apparent, within-cohort AUC of 0.750 (95% CI = 0.718–0.782) and an apparent average precision of 0.594 (Table 2). These metrics describe the model including clinical covariates and treatment arm, rather than the score alone.

**Table 2:** Association of the ECM–immune exclusion score with pCR and selected-model performance.

Dataset	Role	Adjustment	n	pCR	OR (95% CI)	P	AUC (95% CI)	AP
I-SPY2	Main	HR / HER2 / MammaPrint / arm	987	319	0.687 (0.588–0.803)	<0.001	0.750 (0.718–0.782)	0.594
GSE25066	Primary external	ER / stage / source	482	98	0.744 (0.568–0.975)	0.032	0.732 (0.677–0.786)	0.379
GSE20271	Primary external	ER / HER2 / treatment	178	26	0.407 (0.232–0.715)	0.002	0.798 (0.710–0.885)	0.474
GSE20194 all	Auxiliary	ER / HER2	278	56	0.824 (0.578–1.175)	0.285	0.798 (0.729–0.867)	0.514
GSE20194 train+valid	Auxiliary	ER / HER2	230	48	0.764 (0.511–1.144)	0.191	0.821 (0.748–0.895)	0.577
GSE20194 validation	Sensitivity	Univariable	100	15	0.812 (0.464–1.418)	0.463	0.569 (0.421–0.717)	0.182

Notes: ORs are for a 1-SD higher ECM–immune exclusion score. AUC and AP are apparent performance of the separately fitted selected models; optimism-corrected estimates are given separately. The GSE20194 rows are nested and overlapping auxiliary/sensitivity sets, not independent confirmations. pCR, pathological complete response; OR, odds ratio; CI, confidence interval; AUC, area under the receiver operating characteristic curve; AP, average precision; HR, hormone receptor; ER, estrogen receptor; HER2, human epidermal growth factor receptor 2.

Fig 2 summarizes the association estimates and selected-model performance measures.

**Fig 2:**
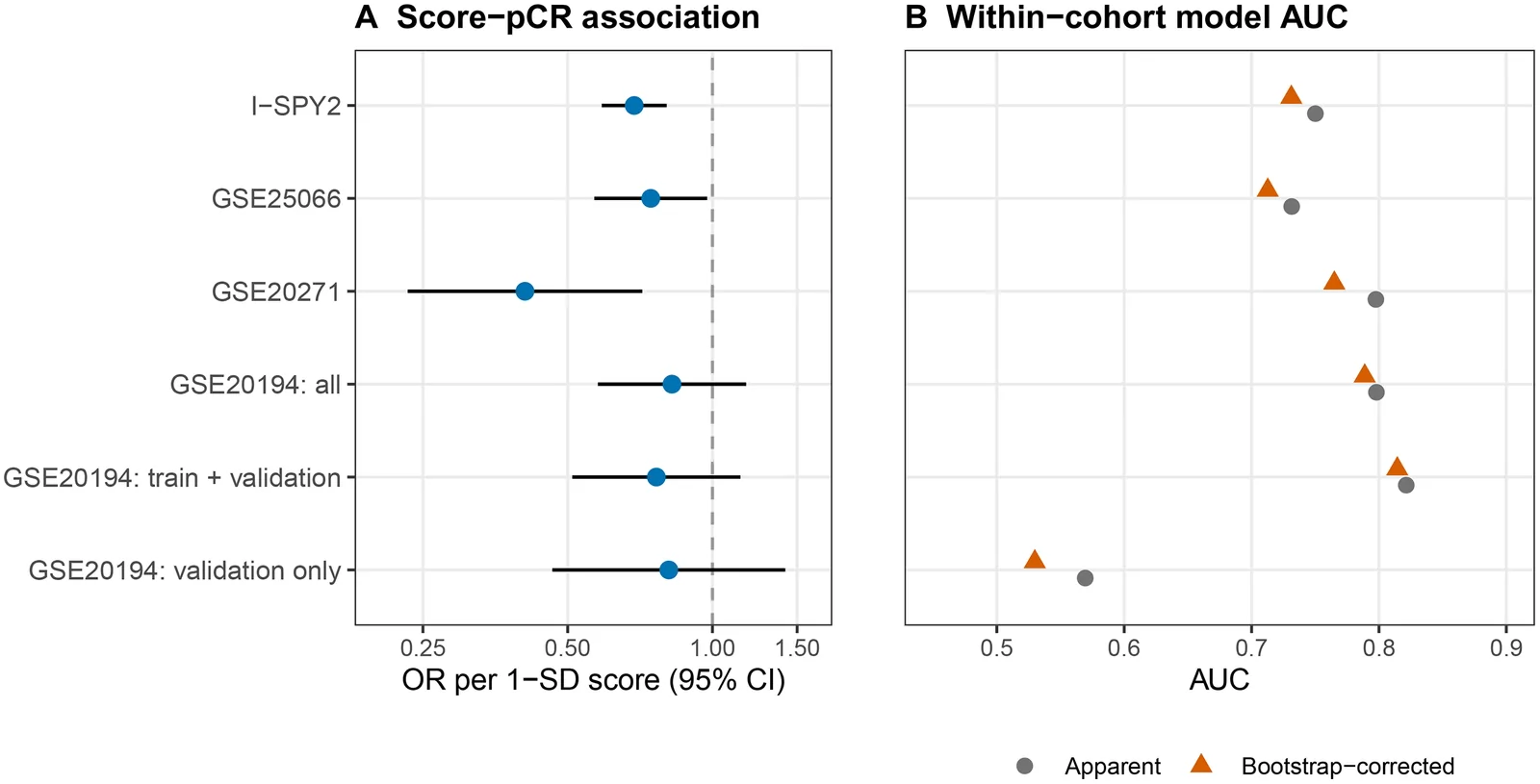
Score associations and within-cohort model performance. (A) Score ORs per standard deviation with 95% confidence intervals, using cohort-specific covariates; the GSE20194 validation-only model is univariable. (B) Apparent and 500-bootstrap optimism-corrected AUC point estimates. Correction is conditional on processed scores and does not validate a locked clinical model. GSE20194 analyses are overlapping auxiliary evidence.

Both primary external cohorts showed the same inverse pattern. In GSE25066, after adjustment for estrogen receptor status, clinical stage, and study source, higher scores were associated with reduced pCR odds (OR=0.744, 95% CI = 0.568–0.975, *P* = 0.032; AUC = 0.732). In GSE20271, after adjustment for estrogen receptor status, HER2 status, and treatment, the score showed a stronger inverse estimate (OR=0.407, 95% CI = 0.232–0.715, *P* = 0.002; AUC = 0.798) (Table 2; Fig 2).

### Shared records across external datasets

Revision-stage auditing identified 187 cross-dataset pairs supported by normalized source labels and expression-profile Spearman correlations exceeding 0.999 across 22,283 common probes. An additional two pairs had matching source labels without corresponding high profile correlations and remain unresolved. All 189 candidate pairs had concordant pCR annotations; 92 of the 100 GSE20194 MAQC validation records overlapped under either definition. No profile correlations above 0.99 were found between GSE20271 and either other external dataset. This screen supports the identified reuse but cannot exclude every form of patient overlap. GSE20194 must not be counted as additional independent validation of GSE25066.

Excluding the 187 pairs supported by both labels and profiles left 91 GSE20194 patients with 25 pCR events (adjusted OR 0.844, 95% CI 0.493–1.445; P = 0.536) and 295 GSE25066 complete cases with 67 events (OR 0.714, 95% CI 0.516–0.988; P = 0.042). A conservative sensitivity excluding all 189 candidates left 89 GSE20194 patients with 24 events (OR 0.834, 95% CI 0.485–1.434) and 293 GSE25066 complete cases with 66 events (OR 0.699, 95% CI 0.504–0.970). Models retained original full-cohort score scaling and preprocessing. Only eight nonshared patients remained in the MAQC validation subset; their counts are reported without fitting a purported independent validation model. All original and exclusion analyses are retained.

### Auxiliary and sensitivity analyses

In the GSE20194 all-sample auxiliary analysis, the ER/HER2-adjusted estimate followed the expected direction without reaching statistical significance (OR=0.824, 95% CI = 0.578–1.175, *P* = 0.285). The univariable model in the smaller validation-only subset also yielded a nonsignificant estimate (OR=0.812, 95% CI = 0.464–1.418, *P* = 0.463). These findings were retained as auxiliary and sensitivity evidence rather than primary validation claims.

### Model performance beyond clinical covariates

Across the selected main and validation models, AUC values ranged from 0.732 to 0.798 in the main and primary external cohorts, and average precision values exceeded cohort-specific pCR prevalence (Fig 2). Adding the score to available clinicopathologic covariates produced modest AUC gains of 0.011 in the I-SPY2 clinical-plus-arm model, 0.023 in GSE25066, and 0.063 in GSE20271. These changes indicate additive information, not a stand-alone clinical decision model. Full model-performance and model-comparison summaries are provided in S3 and S4 Table; exploratory interaction and decision-curve summaries are provided in S5 and S6 Table.

### Bootstrap internal validation

Optimism-corrected AUCs were 0.731 in I-SPY2, 0.713 in GSE25066, and 0.765 in GSE20271, compared with apparent AUCs of 0.750, 0.732, and 0.798. Corrected AUCs were 0.789 and 0.814 in the nested GSE20194 all-sample and training-plus-validation analyses, respectively; these were separate fitted multivariable models, not independent replications. The validation-only univariable analysis had a corrected AUC of 0.530. All 500 regression refits per analysis set completed without recorded fitting warnings or errors, but successful numerical convergence did not ensure stable calibration: bootstrap test calibration slopes were highly variable in the validation-only subset, making the mean-based correction unstable. This result is retained as a limitation rather than interpreted as reliable calibration performance.

### Model diagnostics and calibration limitations

All six selected original model designs were full rank and converged with finite coefficients; the numerical linear-programming screen detected no separating direction. These findings do not remove small-sample uncertainty. The apparent-calibration bootstrap recorded 100 extreme-probability warnings: 98 in GSE20271 and two in the GSE20194 validation-only subset, all during spline calibration rather than prediction-model refitting. All 3,000 prediction-model refits and evaluated curve points returned numerical results, but the calibration bands and unstable small-sample slopes remain exploratory (S3 Table; S1 Fig). Optimism-corrected decision curves are supplied only as illustrative summaries (S6 Table; S2 Fig).

### Penalized and missing-data sensitivity analyses

I-SPY2/GSE194040: penalty 1, OR 0.693 (bootstrap interval 0.584–0.806); penalty 10, OR 0.710 (bootstrap interval 0.616–0.802). GSE25066: penalty 1, OR 0.742 (bootstrap interval 0.578–0.946); penalty 10, OR 0.756 (bootstrap interval 0.630–0.888). GSE20271: penalty 1, OR 0.466 (bootstrap interval 0.260–0.731); penalty 10, OR 0.642 (bootstrap interval 0.479–0.915). GSE20194/validation_only: penalty 1, OR 0.824 (bootstrap interval 0.526–1.284); penalty 10, OR 0.891 (bootstrap interval 0.696–1.145). The all-sample and training-plus-validation GSE20194 analyses showed penalty-dependent intervals; the full results are retained, and the original nonsignificant estimates are not reclassified as successful validation.

In GSE25066, 20 of 508 records had unusable outcome annotations. Among the remaining 488 patients, six lacked the model ER covariate, yielding 482 complete cases with 98 pCR events. A supplementary multiple-imputation analysis used 50 logistic-regression imputations of ER, conditioned on pCR, the score, stage and study source, with 10 iterations and seed 123. Outcomes were not imputed. This analysis assumes missing at random conditional on these variables; the assumption cannot be verified from the observed data. The pooled score OR was 0.752 (95% CI 0.574–0.985); complete pooled coefficient estimates and intervals are retained in the supplementary output.

### Preprocessing and gene-coverage sensitivity

From the public source expression matrices, we reconstructed the original external-cohort scores, with maximum absolute differences below 10^−10^. We compared the original probe-selection rule with selection by native-scale interquartile range, mean or median expression across mapped probes, and within-gene rank-based inverse-normal transformation. Native input scales were retained rather than applying an indiscriminate second logarithm. Across these variants, adjusted ORs ranged from 0.728 to 0.744 in GSE25066, 0.407 to 0.441 in GSE20271, and 0.781 to 0.824 in the GSE20194 all-sample analysis. GSE20194 remained nonsignificant under every evaluated preprocessing rule.

In I-SPY2, removing NLRC5, CD274, or both from the immune component yielded adjusted ORs of approximately 0.687 in each case, compared with 0.687 for the full score. These analyses test the effect of the two unavailable GPL96 genes in the main cohort; they do not establish that the unobserved genes behave equivalently in the external cohorts or validate a universal reference distribution. Platform-level gene coverage also differs from per-sample availability: component means follow the original available-value rule when a sample has missing expression measurements.

### Treatment and receptor sensitivity

Using a common receptor adjustment core (HR and HER2 where variable) retained inverse estimates in I-SPY2, GSE25066 and GSE20271, but not a statistically clear estimate in GSE20194. HR was reconstructed from observed ER and PR annotations in the external datasets: either known positive result defined positivity, while negativity required both known negative results; otherwise HR was missing. These models harmonize receptor definitions but do not eliminate treatment or ascertainment differences.

In GSE20271, adjustment for randomized rather than received treatment group yielded an OR of 0.420 (95% CI 0.242–0.728); excluding seven treatment crossovers yielded an OR of 0.397 (95% CI 0.224–0.704). In the I-SPY2 source-coded Paclitaxel group alone, the HR/MammaPrint-adjusted OR was 0.830 (95% CI 0.541–1.274; n = 179, 31 pCR events). Thus, the association was not statistically clear in every treatment context, and these analyses do not demonstrate differential benefit from a specific regimen.

### RPPA associations with immune-related protein markers

RPPA analyses matched 736 I-SPY2 RPPA-mRNA patients and 139 protein or phosphoprotein markers. We report each marker-specific sample count, Spearman coefficient, nominal P value, Benjamini-Hochberg q value, and a percentile interval from 500 patient bootstrap resamples. These intervals describe cross-sectional correlation uncertainty, not mechanistic validation. Higher ECM-immune exclusion scores correlated negatively with immune-related protein markers, including HLA-DR total (rho = −0.498), PD-L1 (rho = −0.488), CD3 epsilon (rho = −0.352), and STAT1 Y701 (rho = −0.267) (Fig 3). This protein-level pattern was compatible with an immune-low state in high-score tumors.

**Fig 3:**
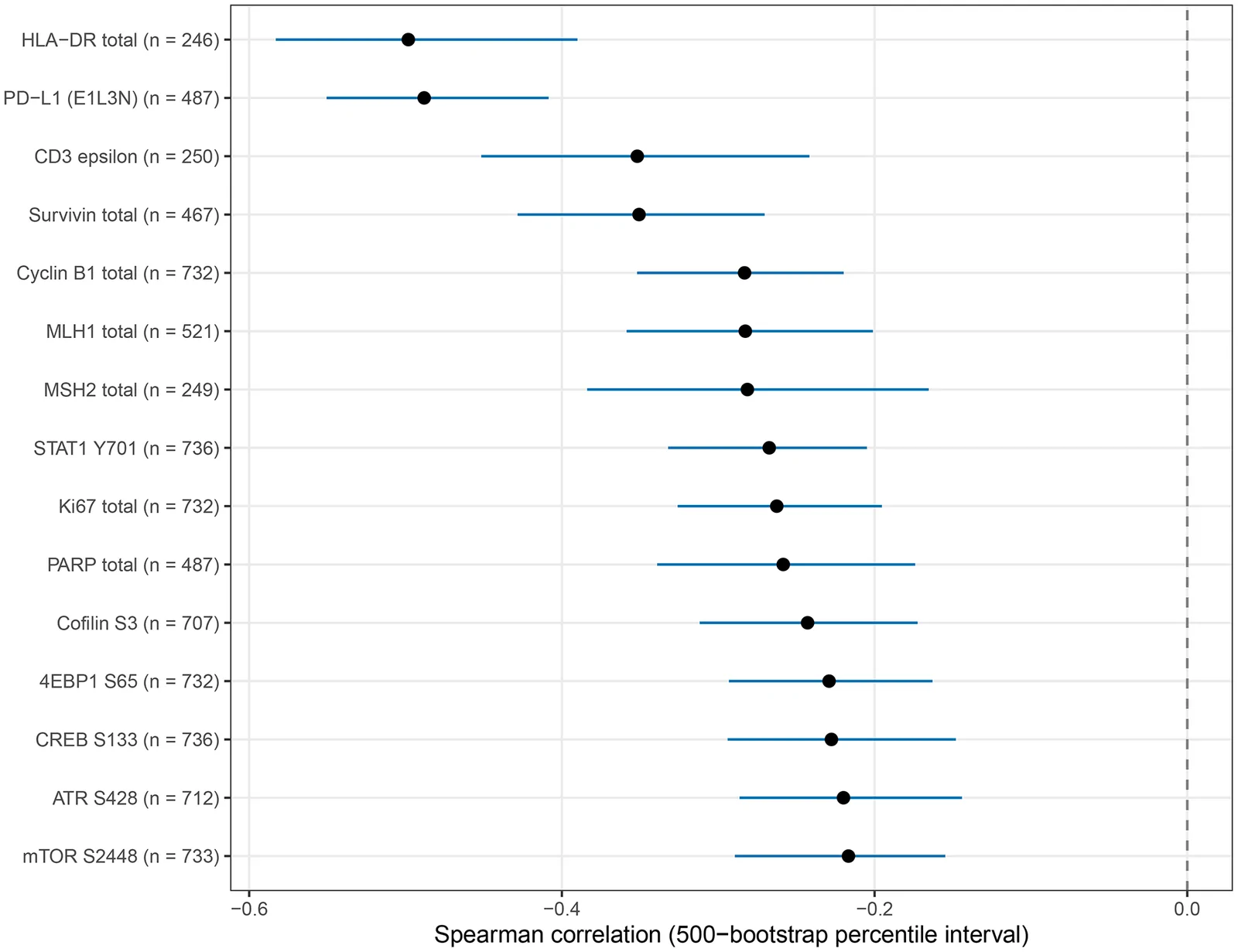
Protein-marker correlations with the transcriptomic score. Points and intervals show the 15 largest absolute Spearman correlations and 500-bootstrap percentile intervals. All 139 markers, per-marker sample counts, nominal P values and BH q values are supplied. Matched samples numbered 736 overall; individual marker counts vary.

### Additional source-definition and eligibility sensitivity

Restriction to the source-defined breast-and-node endpoint sets retained the I-SPY2 and GSE20271 inverse associations, while GSE20194 MAQC230 remained nonsignificant (OR 0.764, 95% CI 0.511–1.144). These are the same cohort-specific fits under a narrower evidence inventory. GSE25066 endpoint-usable annotations include five HER2-positive and 17 unknown records despite HER2-negative eligibility in its source report. We retained the deposited labels and added a known-HER2-negative sensitivity: the complete-case model included 465 patients and 93 pCR events, with OR 0.775 (95% CI 0.590–1.019; *P* = 0.068). The direction persisted but statistical uncertainty increased; the original significant estimate is not uniformly robust to this restriction (S2 Table).

## Discussion

This retrospective analysis found inverse score–pCR associations in I-SPY2, GSE25066 and GSE20271, while GSE20194 estimates were weaker and nonsignificant. The result concerns a bulk-transcriptomic ECM-high/immune-low state. It does not directly measure spatial immune exclusion or demonstrate an ECM-driven causal mechanism. Matched RPPA correlations provide cross-sectional protein context, not mechanistic validation.

The contribution is the evaluation of a simple contrast between biologically motivated expression components across heterogeneous public response datasets. ECM- and immune-related biomarker concepts were already studied; Liu et al., for example, developed a survival-derived ECM index and a response model [9]. The present score was not trained by optimizing gene weights against pCR, but this distinction does not make the entire study prospectively prespecified or eliminate selection and reporting bias. An immutable pre-analysis record of all analytical decisions was not identified, and cohort classification was refined during feasibility assessment.

Record overlap materially limits the apparent number of replications. GSE20194 and GSE25066 share 187 pairs supported by normalized source labels and highly correlated expression profiles, with two additional label-only candidates remaining unresolved. This includes 92 of the 100 MAQC validation records. GSE20194 therefore cannot provide additional independent confirmation of GSE25066. Exclusion analyses retained the GSE25066 association and the nonsignificant GSE20194 estimate, but do not establish independence of every patient in every source cohort. All original and overlap-sensitive results are reported.

The statistical sensitivity analyses refine rather than remove uncertainty. Ridge estimates retained the principal inverse directions, with attenuation in the small GSE20271 cohort under stronger penalization. Multiple imputation of the six missing ER covariates in GSE25066 gave an estimate close to the complete-case result under a conditional missing-at-random assumption. Alternative probe aggregation and rank normalization retained the main pattern, while GSE20194 remained nonsignificant. Restriction to deposited HER2-negative GSE25066 records retained direction but yielded a confidence interval crossing unity, further limiting the strength of external confirmation. These analyses do not replace prospective validation, justify a universal assay cutoff, or eliminate unmeasured confounding.

Treatment context remains consequential. Covariate sets and source treatment regimens differ, so the fitted models do not estimate a universal treatment-independent effect. Randomized-treatment and crossover sensitivities in GSE20271 retained the direction, whereas the I-SPY2 Paclitaxel-only source group did not provide a statistically clear association. The interaction analyses were exploratory, and nominal findings did not survive BH adjustment. These results do not show that the score predicts benefit from choosing one regimen over another.

Apparent AUC values belonged to separately fitted cohort-specific models, not the score alone. Bootstrap-corrected AUCs were lower, and calibration estimates were especially unstable in the GSE20194 validation-only subset. Apparent calibration bands condition on the original fitted probabilities, while the corrected performance and decision-curve estimates condition on processed scores. Illustrative decision thresholds lack a validated clinical action; the curves cannot establish treatment selection or immediate patient-level utility. New-patient application would require an independently specified assay and reference distribution, a locked model, and external calibration.

Additional limitations include source-specific endpoint annotations, including the unavailable I-SPY2 breakdown of administrative non-pCR categories, incomplete clinical stage and treatment information, heterogeneous pretreatment specimen types, platform-dependent coverage, and per-sample missing expression values. Fine-needle aspirates may enrich neoplastic cells and underrepresent stromal compartments, and the GSE20271 source description includes primary-tumor or axillary-metastasis samples. These features can change an ECM/immune expression contrast without demonstrating a change in spatial immune exclusion. Available retrospective bulk data cannot establish cellular localization, prospective performance, or causality.

The score should therefore be regarded as a hypothesis-generating transcriptomic biomarker associated with pCR in the principal fitted cohorts, with attenuated evidence in overlapping GSE20194 analyses. Further studies should lock preprocessing and score calculation, resolve assay and sampling effects, and test calibration and clinical decisions prospectively. Spatial and experimental studies would be required to evaluate the proposed biological interpretation.

## Supporting information

S1 Table Biologically motivated ECM/CAF/TGF-β and IFN-γ/cytotoxic/antigen-presentation gene sets. (TSV)

S2 Table Cohort source characteristics, endpoint-value mapping, missingness, clinical distributions, RCB cross-check and source-definition/eligibility sensitivity analyses. (ZIP)

S3 Table Full model coefficients, apparent performance, internal validation and numerical diagnostics. (ZIP)

S4 Table Model-comparison summaries for the addition of the ECM-immune exclusion score. (ZIP)

S5 Table Exploratory score-by-factor interaction tests. (ZIP)

S6 Table Exploratory decision-curve key-threshold summaries. (ZIP)

S7 Dataset Machine-readable numerical data underlying the main and supplementary figures, including all 139 RPPA markers. (ZIP)

S8 Code R and Python scripts, raw-input and output manifests, and instructions for reproducing cohort flow, score calculation, models, RPPA results, interactions, sensitivity analyses and figures. (ZIP)

S9 Table Analysis inventory and complete sensitivity results for penalization, imputation, preprocessing, overlap, treatment/receptors and duplicate-platform selection. (ZIP)

S1 Fig Apparent calibration curves with conditional pointwise bootstrap bands. Bands do not account for full model development; small-sample spline instability is reported.
(PDF)

S2 Fig Illustrative decision curves with optimism-corrected net-benefit estimates. Thresholds do not correspond to a validated treatment-selection action.
(PDF)

S3 Fig Distributions and relationships of the ECM and immune components by cohort and deposited platform. All scored expression records are shown, including both records of the duplicated I-SPY2 patient; this descriptive analysis does not establish cross-platform interchangeability.
(PDF)
