# Supplementary material for: A pretreatment ECM-high/immune-low transcriptomic score is associated with pathological complete response after neoadjuvant therapy in breast cancer
Source: PLoS One. 2026 Nov 5;21(11):e0357532. doi: 10.1371/journal.pone.0357532 (PMC13739100; doi:10.1371/journal.pone.0357532)
Supplement: Supplementary file 1 [file pone.0357532.s010.pdf]

Apparent calibration with pointwise uncertainty

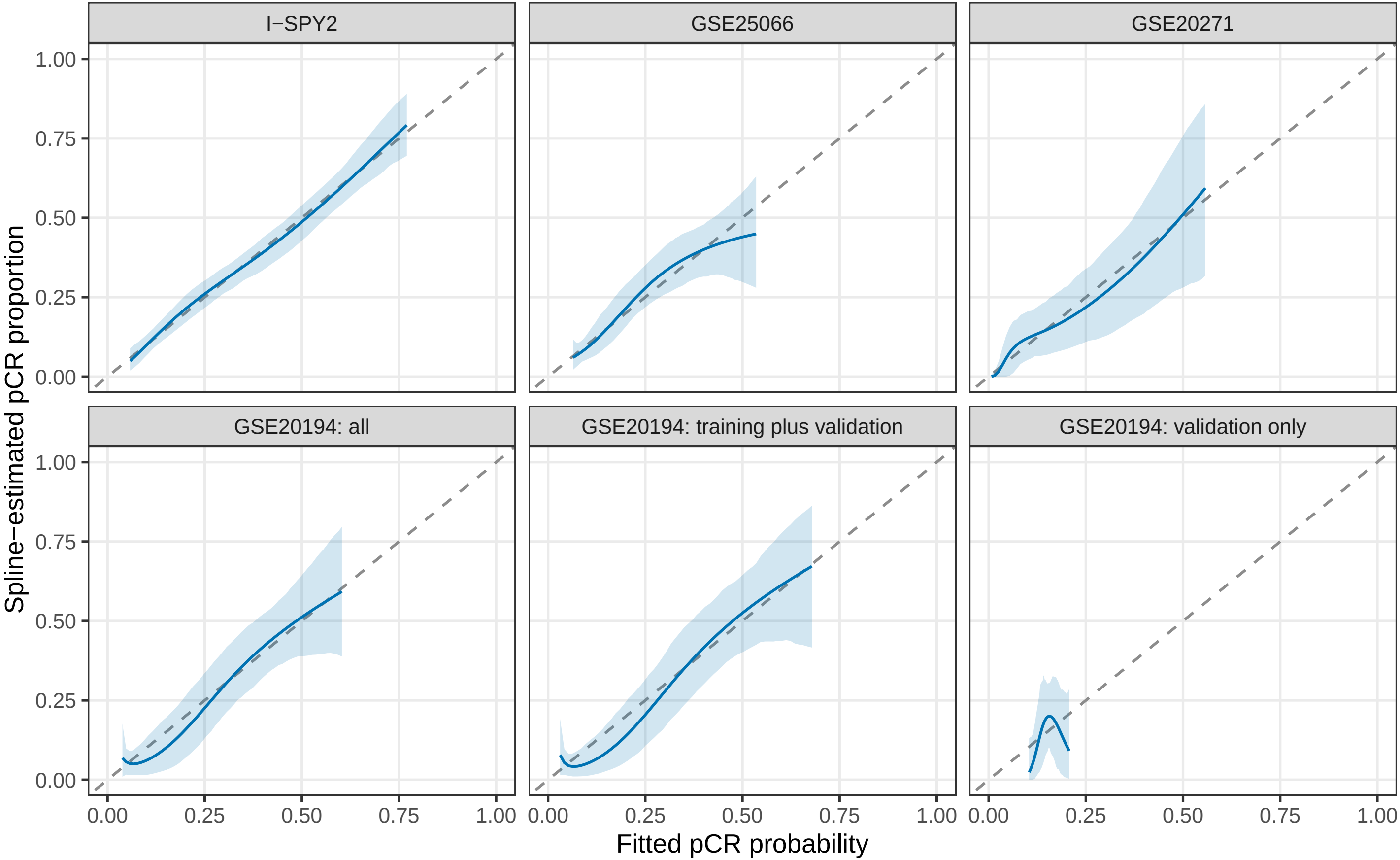

Bands: 95% pointwise patient−bootstrap intervals conditional on the original fitted probabilities.
Curves are apparent, not externally validated or optimism−corrected. Display limited to 2.5th−97.5th prediction percentiles.
Bootstrap−corrected AUC/Brier/slope estimates are reported separately; GSE20194 subsets overlap and are nested.
