# Supplementary material for: A pretreatment ECM-high/immune-low transcriptomic score is associated with pathological complete response after neoadjuvant therapy in breast cancer
Source: PLoS One. 2026 Nov 5;21(11):e0357532. doi: 10.1371/journal.pone.0357532 (PMC13739100; doi:10.1371/journal.pone.0357532)
Supplement: Supplementary file 2 [file pone.0357532.s011.pdf]

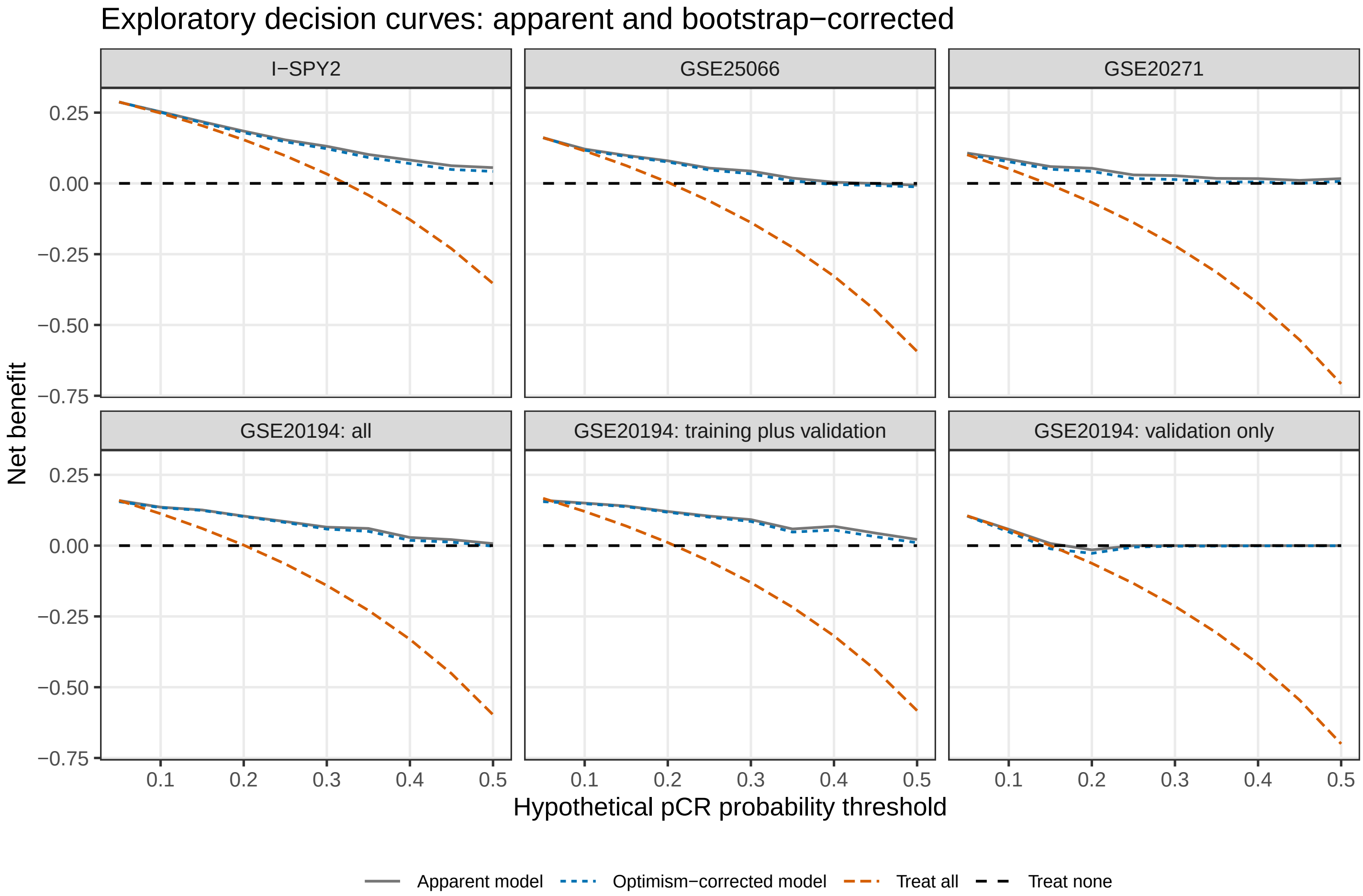

Thresholds 0.05−0.50 are illustrative; no validated treatment action or clinically justified threshold is specified.
Treat−all/treat−none are mathematical comparators for a hypothetical action in likely responders.
Curves cannot establish treatment−selection benefit; correction is conditional on processed scores.
