# Supplementary material for: A pretreatment ECM-high/immune-low transcriptomic score is associated with pathological complete response after neoadjuvant therapy in breast cancer
Source: PLoS One. 2026 Nov 5;21(11):e0357532. doi: 10.1371/journal.pone.0357532 (PMC13739100; doi:10.1371/journal.pone.0357532)
Supplement: Supplementary file 3 [file pone.0357532.s012.pdf]

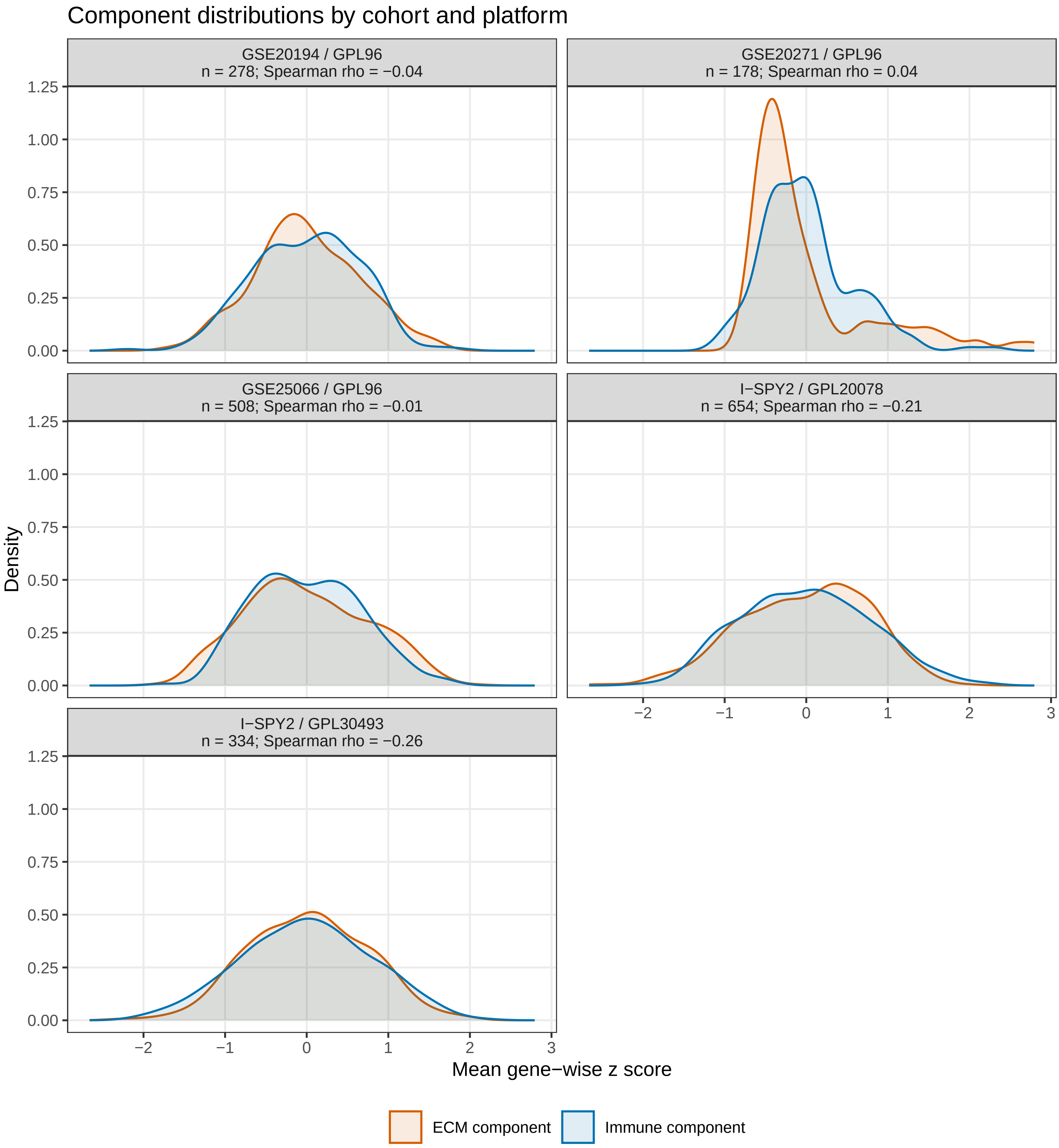

All scored expression records; I−SPY2 includes both records of its one duplicated patient.
Components are means of available gene−wise z scores, before component standardization.
I−SPY2 platform groups share the original normalization; external cohorts use their own normalization.
Distributions and correlations are descriptive and do not establish assay interchangeability.

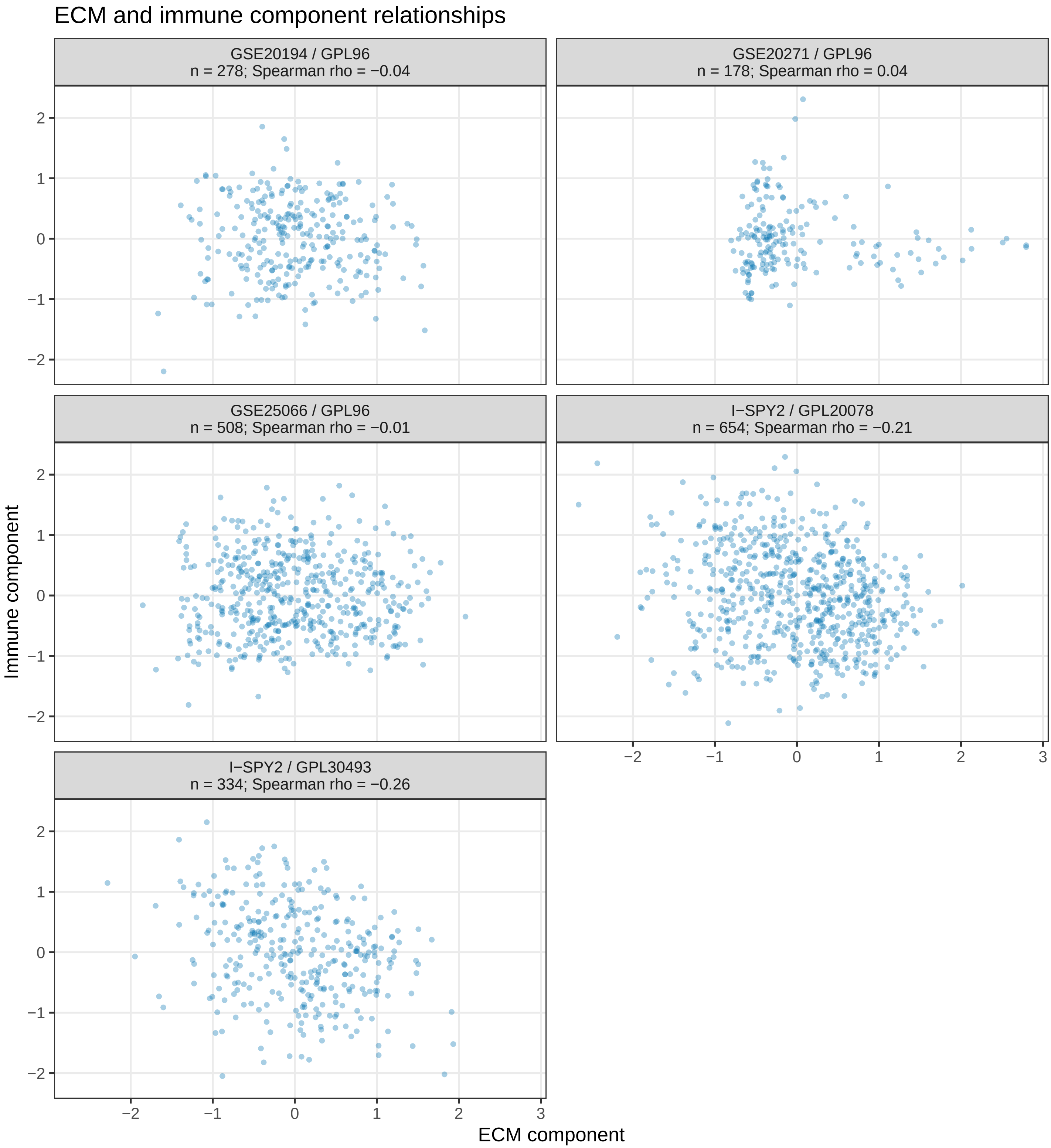

All scored expression records; I−SPY2 includes both records of its one duplicated patient.
Components are means of available gene−wise z scores, before component standardization.
I−SPY2 platform groups share the original normalization; external cohorts use their own normalization.
Distributions and correlations are descriptive and do not establish assay interchangeability.
